# Urea Is Both a Carbon and Nitrogen Source for *Microcystis aeruginosa*: Tracking ^13^C Incorporation at Bloom pH Conditions

**DOI:** 10.3389/fmicb.2019.01064

**Published:** 2019-05-17

**Authors:** Lauren E. Krausfeldt, Abigail T. Farmer, Hector F. Castro Gonzalez, Brittany N. Zepernick, Shawn R. Campagna, Steven W. Wilhelm

**Affiliations:** ^1^Department of Microbiology, The University of Tennessee, Knoxville, Knoxville, TN, United States; ^2^Department of Chemistry, The University of Tennessee, Knoxville, Knoxville, TN, United States

**Keywords:** HABs, cyanobacteria, Lake Erie, nitrogen, stable isotope probing

## Abstract

The use of urea as a nitrogenous fertilizer has increased over the past two decades, with urea itself being readily detected at high concentrations in many lakes. Urea has been linked to cyanobacterial blooms as it is a readily assimilated nitrogen (N) - source for cyanobacteria that possess the enzyme urease. We tested the hypothesis that urea may also act as a carbon (C) source to supplemental growth requirements during the alkaline conditions created by dense cyanobacterial blooms, when concentrations of dissolved CO_2_ are vanishingly low. High rates of photosynthesis markedly reduce dissolved CO_2_ concentrations and drive up pH. This was observed in Lake Erie during the largest bloom on record (2015) over long periods (months) and short periods (days) of time, suggesting blooms experience periods of CO_2_-limitation on a seasonal and daily basis. We used ^13^C-urea to demonstrate that axenic cultures of the model toxic cyanobacterium, *Microcystis aeruginosa* NIES843, assimilated C at varying environmentally relevant pH conditions directly into a spectrum of metabolic pools during urea hydrolysis. Primarily, ^13^C from urea was assimilated into central C metabolism and amino acid biosynthesis pathways, including those important for the production of the hepatotoxin, microcystin, and incorporation into these pathways was at a higher percentage during growth at higher pH. This corresponded to increased growth rates on urea as the sole N source with increasing pH. We propose this ability to incorporate C from urea represents yet another competitive advantage for this cyanobacterium during dense algal blooms.

## Introduction

Harmful cyanobacterial blooms are expanding globally across freshwater lakes and drinking water reservoirs. *Microcystis*-dominated blooms are especially prevalent and have been detected now on six continents (Harke et al., 2016). Blooms are commonly associated with severely impaired water quality and the production of the class of cyanotoxins, the microcystins: secondary metabolites originally known as “*Fast Death Factor*” (Bishop et al., 1959). These compounds act as potent hepatotoxins to animals and humans, thus directly impact public health and the economy (Bullerjahn et al., 2016; Carmichael and Boyer, 2016). Nutrient overloading – specifically phosphorus (P) and nitrogen (N) – is widely accepted as a primary cause of cyanobacterial blooms in freshwater ecosystems (EPA, 2015; Paerl et al., 2016). N inputs in particular are becoming a major concern due the drastic global increase in fertilizer consumption over the last several decades, including the United States (Glibert et al., 2014; Cao et al., 2017). Indeed, increased N loads have been linked to bloom formation and the production of microcystins (Gobler et al., 2016; Paerl et al., 2016). Yet, while researchers commonly agree that nutrient concentrations can constrain plankton biomass, less information is available about the factors that result in specific biological species compositions.

The use of urea (CH_4_N_2_O) as a nitrogenous fertilizer has increased ∼100-fold in the last four decades, and now makes up >50% of the total N fertilizer consumption worldwide (Glibert et al., 2006; Paerl et al., 2016). Residual urea in soils that is not utilized by plants has historically been thought to be consumed by the terrestrial microbial community via the enzyme urease, where the byproduct ammonium can be oxidized to nitrate for denitrification or volatilized to NH_3_ and lost to the atmosphere (Glibert et al., 2014). Yet various agricultural practices, such as the application of urease inhibitors to soils, the timing of fertilizer application before rainfall or prior to snow melt and the broad use of tile drainage systems, increase the likelihood that urea may be exported to nearby aquatic systems (Glibert et al., 2006). Consequently, urea can reach concentrations as high as 150 μM and represent over 50% of the total dissolved N pool, especially in systems proximal to heavy agricultural areas (Bogard et al., 2012; Chaffin and Bridgeman, 2013; Glibert et al., 2014). Consequently, urea has been suggested to contribute to the eutrophication of inland and coastal waters (Finlay et al., 2010; Glibert et al., 2014), and its usage has corresponded to a rise in reports of harmful cyanobacteria in high fertilization areas (Glibert et al., 2014; Paerl et al., 2016). These observations are supported by the field studies demonstrating cyanobacteria can consume urea as a N source, and in some cases preferred urea over oxidized forms of N such as nitrate (Davis et al., 2010; Chaffin and Bridgeman, 2013; Belisle et al., 2016). Studies in culture confirmed these observations and suggest a specific role for urea in toxicity and genomic evolution of major bloom-forming cyanobacterial taxa (Steffen et al., 2014b; Peng et al., 2018).

**FIGURE 1:**
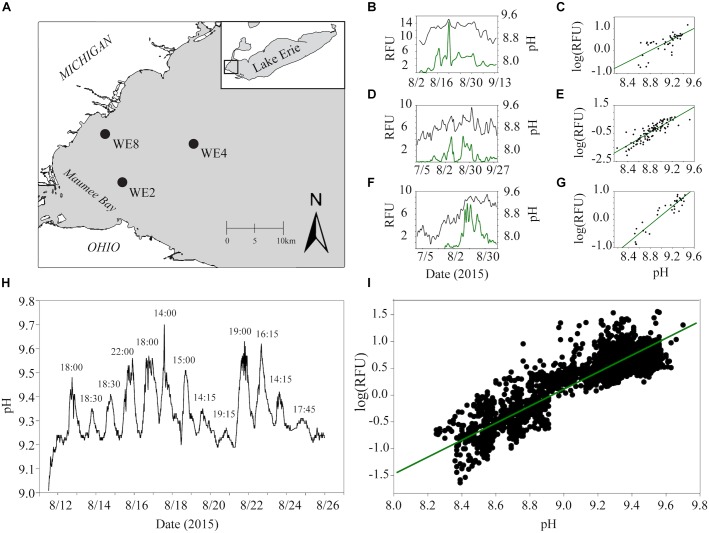
Estimates of real-time phycocyanin concentrations (relative fluorescence units, RFU) and pH data from monitoring buoys in the western basin of Lake Erie were accessed through the NOAA Great Lakes Environmental Research Laboratory website (https://www.glerl.noaa.gov/res/HABs_and_Hypoxia/rtMonSQL. php, accessed March 22, 2018). Data were collected at 15-min intervals for buoys deployed in 2015 at station WE2 and WE4 from July to September and WE8 from August to September which captured before, during and after one of the biggest blooms on record in Lake Erie (Ho and Michalak, 2017). Data for phycocyanin (manufacturers reported resolution ± 0.01 RFU) and pH ( ± 0.01 units) were taken from the onboard EXO2 Multiparameter Sonde averaged over each calendar day for analysis to account for diel variation. Locations of each buoy used for data analysis are shown in black circles in the top left panel **(A)**. Trends in average phycocyanin (relative fluorescence units, RFU) and pH over the course of the summer of 2015 for WE8 **(B)**, WE4 **(D)**, and WE2 **(F)** are shown in the top middle panels. Correlations between average daily phycocyanin and pH for WE8 **(C)**, WE4 **(E)**, and WE2 **(G)** are shown in the top right panels. Changes in pH observed at 15 min intervals for the 2 weeks and Spearman correlation between pH and RFU over these 2 weeks are shown **(H,I)**.

While the importance of urea as a N source to cyanobacteria is becoming more established, urea as a carbon (C) source has been suggested but not directly examined. This source of C may be significant during dense blooms when the dissolved CO_2(aq)_ is drawn down by rampant photosynthesis. CO_2(aq)_-limitation can manifest during the course of a bloom, a condition observable by the proxy of increasing pH (Paerl and Huisman, 2009; Visser et al., 2016; Ji et al., 2017). This trend was clearly demonstrated in the western basin of Lake Erie during the 2015 *Microcystis* bloom (Figure 1). While average daily pH and phycocyanin concentrations were variable across the summer months, both metrics were lower earlier in the summer (July) and gradually increased throughout August and September (Figure 1B,D,F). The relationship between cyanobacterial abundance and pH was strongly and positively correlated, implying that the pH increased in parallel with cyanobacterial abundance. The strongest correlation was observed at station WE2 located near the mouth of Maumee Bay (*R*^2^ = 0.87 at WE2; *R*^2^ = 0.75 at WE4; *R*^2^ = 0.54 at WE8; Figure 1C,E,G), a catchment for the Maumee river which is responsible for the greatest agricultural nutrient loads to the western basin (Michalak et al., 2013). During this period, the average daily pH in the western basin ranged from ∼8.2 to 9.5, and peak phycocyanin values were observed at average pH values of 9.2 and higher. These parameters also varied over the course of the day (Figure 1H) and strongly correlated (*R*^2^ = 0.80; Figure 1I), with the highest pH values and phycocyanin concentrations consistently observed in the late afternoon.

When conditions reach such alkaline levels in lakes, the resulting inorganic C pool is primarily composed of bicarbonate (HCO_3_) and carbonate (CO_3_) (Supplementary Figure S1; Wetzel, 2001). Cyanobacteria are well equipped to handle low CO_2(aq)_ concentrations through the use of highly efficient C concentrating mechanisms (Price, 2011; Visser et al., 2016), although these enzymes require reducing power or ATP. Since the breakdown of urea by urease occurs intracellularly, the CO_2_ from urea hydrolysis should be accessible for C-fixation and as a supplemental C source (Finlay et al., 2010; Sandrini et al., 2014). Indeed, while some may have historically thought of the CO_2_ produced by urea hydrolysis as a waste product (Kandeler et al., 2011), others have hypothesized that it might serve as a C source to phototrophs (Glibert et al., 2016; Steffen et al., 2017). Urea as a C source may especially be relevant during periods of CO_2(aq)_-limitation during dense blooms.

To address the above observations, we investigated the effects of pH (as a control for inorganic C speciation) on the model toxic cyanobacterium *M. aeruginosa* during growth on urea, nitrate or ammonium as sole sources of N. Axenic cell growth was established across a range of environmentally relevant pH conditions that mimicked progression of the bloom season. In culture experiments, ^13^C-labeled urea was used to trace C released during enzymatic hydrolysis and determine whether this cyanobacterium was capable of incorporating this C source.

## Materials and Methods

### Growth of *Microcystis aeruginosa* Cultures on Different N Sources Across pH Conditions

Stock cultures of axenic *Microcystis aeruginosa* NIES843 were maintained in 25 mL of modified CT medium (briefly, 0.05 g Na_2_⋅β-glycerophosphate, 0.04 g MgSO_4_⋅7H_2_0, and trace metals, buffered with 0.2 g TAPS (Steffen et al., 2014b) at a pH of 8.2 with 0.595 mM N (nitrate provided as KNO_3_/CaNO_3_.4H_2_O, ammonium provided as NH_4_Cl or urea) in 50 mL screw cap glass culture tubes. After adjustment with NaOH to change the pH, Na^+^ concentrations totaled ∼1 mM at 8.2. The cultures were incubated without shaking at 26°C on a diel cycle (12:12 h) within the range of 50–60 μmol photon m^-2^ s^-1^ of light. Culture tubes were inverted three times daily, and caps were loosened to allow for gas exchange. Microbial contamination checks were performed regularly by microscopy as well as for heterotrophic microbial growth in purity tubes of both rich and minimal media (LB and CT + 0.05% glucose, 0.05% acetate, 0.05% pyruvate, and 0.05% lactate, respectively). Total dissolved inorganic C (DIC) concentrations in the media after autoclaving, cooling and acclimation to 26°C were measured at the Water Quality Core Facility at the University of Tennessee using a Carbon/Nitrogen Analyzer (Shimadzau TOC-L_CSH_). DIC concentrations of the medium were an average of 4.3 mg/L regardless of inversion.

Prior to starting each growth curve, cells were collected on a 1.0-μm nominal pore-size polycarbonate filter and resuspended into experimental media at a final pH of 7.7 ± 0.05, 8.2 ± 0.05, 8.7 ± 0.05 or 9.2 ± 0.05 (adjusted with NaOH after autoclaving) for each respective N source. Cell concentrations were measured using flow cytometry (Guava easyCyteHT, Millipore) and were used as an inoculum. Growth curves were performed at a starting concentration of ∼75,000 cells/mL, and chlorophyll *a* autofluorescence (fluorescence signal units, FSU) was measured using a fluorometer (Turner Designs TD-700) over the course of the experiment. Samples were examined at approximately the same time every day for consistency after mixing by inversion. Cell concentration and FSU from preliminary growth curves performed the same way on different N sources correlated strongly and significantly (*R*^2^ = 0.96, *p* = 0.0001; Supplementary Figure S2) demonstrating that FSU serves as a suitable proxy for cell density. The additional Na^+^ added to achieve higher pH media conditions (at pH 9.2, there was a total of ∼1.7 mM Na^+^) did not have an influence on growth rate or biomass accumulation (Supplementary Figure S3). These experiments were performed by adding NaCl to media at a pH of 8.2 so that the total Na^+^ concentration matched media at pH of 9.2 that was adjusted with a larger amount of NaOH. To determine if the cells were limited by C in the above conditions, *M. aeruginosa* was grown on nitrate with 0.238 mM Na_2_HCO_3_ at a pH of 8.2 and 9.2.

### Tracing Cellular Metabolites and ^13^C Incorporation From Urea

Axenic *M. aeruginosa* NIES 843, acclimated to growth on ^12^C-urea, was transferred to media containing saturated (>99%) ^13^C-urea as the sole N source at a starting concentration of 0.585 mM N in 25 mL of modified CT medium adjusted to three different pH values using NaOH. These three values were chosen to capture a range of pH that would be observed prior to a bloom, during a bloom, and at the late stages of a bloom. For each condition, the pH was empirically determined at the onset of inoculation (after autoclaving media, which can alter pH) and resulted in pH of 7.5 ± 0.2, 8.4 ± 0.2 and 9.5 ± 0.2 for experimental triplicates. The approximate proportions of DIC in each treatment can be referenced in Supplementary Figure S1. Cultures were inoculated at ∼25 FSU (∼75,000 cells/mL) and grown for 7 days. On day 7, cell concentration and FSU were measured (Supplementary Table S1), each culture was filtered onto a 1.0-μm nominal pore-size polycarbonate filter, and metabolites were immediately extracted at 4°C with pre-chilled extraction solvent (1.3 mL of 40:40:20 HPLC grade ACN/MeOh/H_2_O with 0.1 M formic acid). Sample 8.4 R2 did not grow and was excluded from the study.

Extracted metabolites were stored at -20°C until being dried under a stream of N_2_ and resuspended in sterile water. Samples were immediately placed in the Ultimate 3000 RS autosampler (Dionex, Sunnyvale, CA) and an injection volume of 10 μL was separated through a Synergi 2.5 μm Hyrdo-RP, 100 Å, 100 × 2.00 mm liquid chromatography column (Phenomenex, Torrance, CA, United States) kept at 25°C. The mass spectrometer was run in full scan mode following a protocol adapted from Rabinowitz (Lu et al., 2010). The chromatographic eluent was ionized via an electrospray ionization (ESI) source in negative mode and coupled to an Exactive Plus Orbitrap mass spectrometer (Thermo Fisher Scientific, Waltham, MA, United States) through a 0.1-mm internal diameter fused silica capillary tube. The samples were run with a spray voltage of 3 kV, N sheath gas of 10 (arbitrary units), capillary temperature of 320°C, and automatic gain control (AGC) target set to 3 × 10^6^ ions. Samples were analyzed at a resolution of 140,000 and a scan window of 85–800 m/z from 0 to 9 min and 110–1000 m/z from 9 to 25 min. Solvent A consisted of 97:3 water:methanol, 10 mM tributylamine, and 15 mM acetic acid. Solvent B was 100% methanol. The solvent gradient from 0 to 5 min was 100% A 0% B, from 5 to 13 min was 80% A 20% B, from 13 to 15.5 min was 45% A 55% B, from 15.5-19 min was 5% A 95% B, and from 19 to 25 min was 100% A 0% B with a flow rate of 200 μL/min. Files generated by Xcalibur (RAW) were converted to the open-source mzML format (Martens et al., 2011) via the open-source msconvert software as part of the ProteoWizard package (Chambers et al., 2012). Maven (mzroll) software, Princeton University (Melamud et al., 2010; Clasquin et al., 2012) was used to automatically correct the total ion chromatograms based on the retention times for each sample (Melamud et al., 2010; Clasquin et al., 2012). Metabolites were manually identified and integrated using known masses ( ± 5 ppm mass tolerance) and retention times (Δ ≤ 1.5 min). ^12^C- and ^13^C-metabolites were manually selected and integrated by exact mass ( ± 5 ppm) and known retention time to calculate ^13^C-incorporation (Lu et al., 2010). The estimated natural abundances of ^13^C for metabolites were calculated using the website https://www.sisweb.com/mstools/isotope.htm, and estimated to be within 1% of the actual value. Briefly, isotopic distributions (which take into account known natural abundances, mass and chemical formula for all isotopic variations) were used to calculate the expected percent abundance of the ^13^C isotope for a specific metabolite, which will change depending on the number of carbons. The numbers were reported as normalized values (relative abundances) to the most abundant ion for that metabolite (i.e., ^12^C isotope), so these relative values had to be calculated back to absolute expected percentage (relative abundance of ^13^C divided by the total relative abundance) manually. These were the values reported.

### Data Analysis

Growth rates of *M. aeruginosa* on nitrate, ammonium, and urea at different pH values were calculated in log-linear growth determined by log scaled FSU. Statistical differences in growth on different N-forms across pH treatments were determined using a Two-way ANOVA with *post hoc* pairwise comparisons using the Tukey Multiple Comparison test. To analyze global metabolic trends, total abundances for each metabolite were calculated for average technical duplicates (^12^C + ^13^C normalized by cell number). For NMDS analysis, abundances were log transformed and clustered using Bray Curtis similarity in Primer-e v7 (Clarke and Gorley, 2006). Percent incorporation was determined from ^13^C:^12^C ratios using all of the isotopomers and isotopologs for each individual metabolite. Statistical differences between total abundances of metabolites and percent ^13^C incorporation for each metabolite were determined by One-way ANOVA followed by pairwise comparisons using the Tukey Multiple Comparisons test.

Metatranscriptomes from Lake Erie during the 2014 Toledo water crisis (Steffen et al., 2017) at stations WE2, WE4, and WE8 were evaluated for the expression of the urease alpha subunit (*ureC*) and carbamoyl phosphate synthase large subunit (*carB*). Contiguous sequences (contigs) annotated as *ureC* or *carB* by the SEED database were downloaded along with their taxonomic classifications from RefSeq using the MG-RAST server^1^. Quality controlled reads (Steffen et al., 2017) from the original samples were recruited to the contigs, and recruited reads to each contig were normalized by contig length and library size to identify proportional expression of these genes in environmental samples. Transcriptomes previously generated from *Microcystis aeruginosa* NIES843 (Steffen et al., 2014b) grown in modified CT media with urea, nitrate and ammonium were analyzed for expression of C concentrating genes. Reads were downloaded from the National Biotechnology Information Center small read archive (PRJNA229846). Raw reads were imported into CLC Genomics Workbench and trimmed with a quality limit of 0.05, allowing for two ambiguous base pairs. Trimmed reads were recruited to the *Microcystis aeruginosa* NIES843 genome at a similarity and length fraction of 0.9 for the calculation of gene expression (TPM).

## Results

**FIGURE 2:**
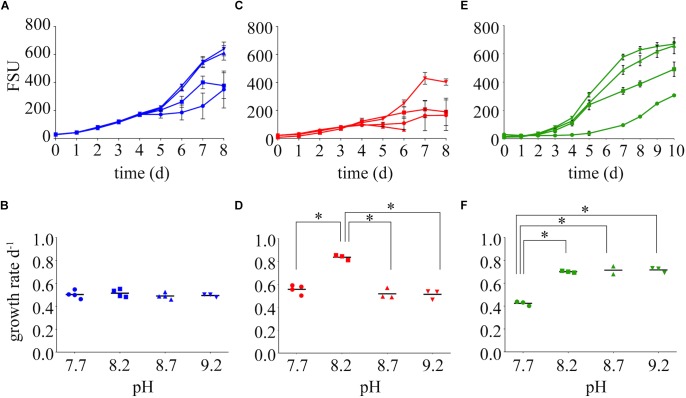
Growth dynamics on nitrate **(A)**, ammonium **(C)**, and urea **(E)** as the sole N source at varying pH values (7.7, 8.2, 8.7, and 9.2). Growth rates are presented below each growth curve for nitrate **(B)**, ammonium **(D)**, and urea **(F)**. Stars represent = statistical significance of differences between growth rates within that N treatment: ^∗∗∗∗^*p* < 0.0001. All *p*-values generated from Two-Way ANOVA can be found in Supplementary Table S2. FSU is a measure of chlorophyll *a* autofluorescence (fluorescence signal units).

Differences in the growth rates of *M. aeruginosa* were driven by both the chemical species of available N and the pH of the growth medium (Figure 2, Supplementary Table S2). Growth rates on nitrate were not affected by pH (Figure 2A,B). The highest growth rate was observed on ammonium at a pH of 8.2 (Figure 2C,D), but this growth rate was not sustained at higher or lower pH values. The next highest growth rate was on urea at 8.2, and this was maintained at an elevated pH of 8.7 and 9.2 (Figure 2E,F). Despite differences in growth among N treatments, the highest pH values supported the highest FSU values by the end of the growth curve for each N treatment, with urea and nitrate reaching an FSU greater than 600 and an average FSU on ammonium reached greater than 400 (Figure 2A,C,E).

**FIGURE 3:**
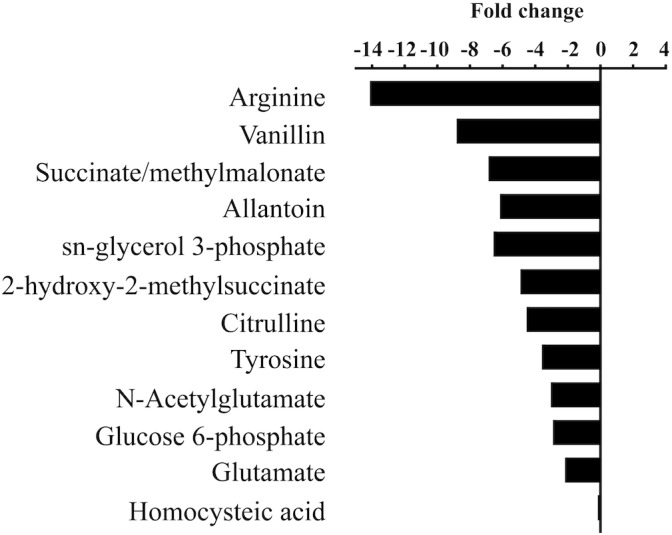
Statistically significant fold changes (*p* < 0.05) in abundances of metabolites in cells grown at high pH (9.5) compared to low pH (7.5).

To determine if *M. aeruginosa* assimilated C from urea during its hydrolysis, metabolites from cells grown on ^13^C-urea were examined by UPLC-HRMS. Cultures were grown at three different pH values to capture the range of conditions that varied in DIC composition (7.5, 8.4, and 9.5). Global metabolite profiles differed between pH treatments (Supplementary Figure S4). The greatest differences were observed in relative abundances of metabolites between the low pH (7.5) and high pH (9.5, Figure 3, Supplementary Figure S5). In general, metabolites normalized by cell number were significantly lower in abundance in cells grown at high pH (Figure 3, Supplementary Figure S5). Significant fold change differences were the greatest for arginine, and significant fold changes were also observed in metabolites important for arginine biosynthesis. Additionally, intermediates in central C metabolism were significantly reduced in cells grown at high pH. While glutamine was not statistically different across treatments of varying pH, glutamate was more abundant at the lowest pH. However, significant differences in the glutamate:glutamine ratio of cultures of varying pH were not observed (*p* = 0.44).

**FIGURE 4:**
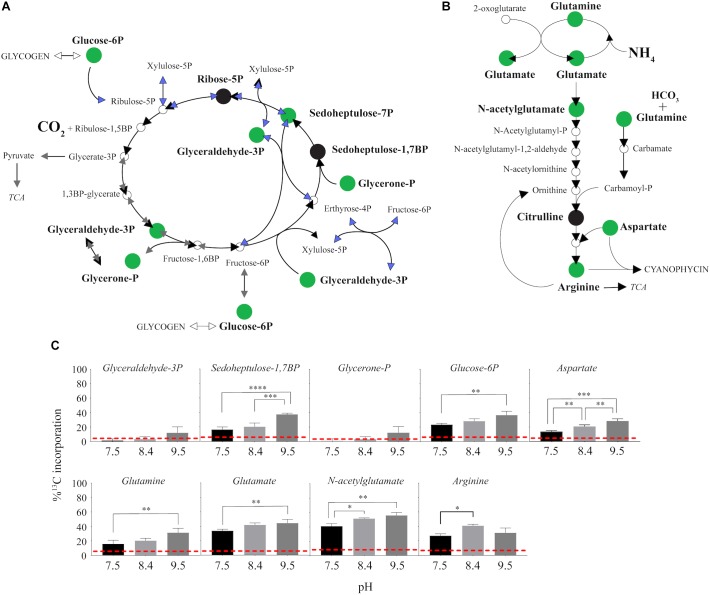
^13^C incorporation from urea into the pathways of central carbon metabolism and pathway for arginine biosynthesis in *M. aeruginosa.* **(A)** The Calvin Cycle (black arrows) with the intersecting glycolysis (gray arrows) and pentose phosphate pathways (blue arrows) adapted from White (2000) and Knoop et al. (2010). **(B)** The classical arginine biosynthesis pathways as adapted from Wendisch (2007). In both **(A)** and **(B)**, the green filled circle indicates a metabolite in which a ^13^C signature was detectable, and black filled circles indicate where the metabolite was detected but with no ^13^C incorporation. Open circles or no circle indicates this metabolite was not detectable. **(C)** Percent incorporation of each metabolites with detectable ^13^C signatures, relative to pH treatment. Red dashed lines represent predicted natural ^13^C abundance, and error bars represent one standard error. Stars represent statistical significance differences between ^13^C incorporation percentages across pH treatments: ^∗^*p* < 0.01; ^∗∗^*p* < 0.05; ^∗∗∗^*p* < 0.001; ^∗∗∗∗^*p* < 0.0001. All *p*-values generated from One-Way ANOVA can be found in Supplementary Table S3.

**FIGURE 5:**
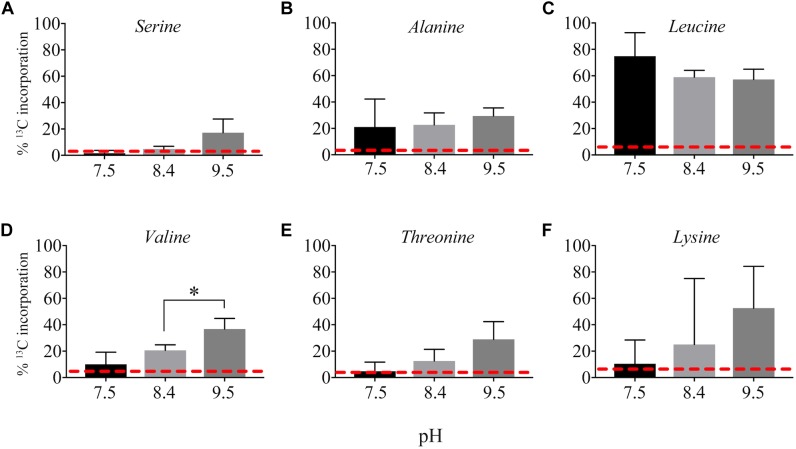
Incorporation of ^13^C from urea into amino acids: serine **(A)**, alanine **(B)**, leucine **(C)**, valine **(D)**, threonine **(E)**, and lysine **(F)**. Red dashed lines represent predicted natural ^13^C abundance, and error bars represent one standard error. Stars represent statistically significant differences in ^13^C incorporation percentages across pH treatments: ^∗^*p* < 0.01. All *p*-values generated from One-Way ANOVA can be found in Supplementary Table S3.

**FIGURE 6:**
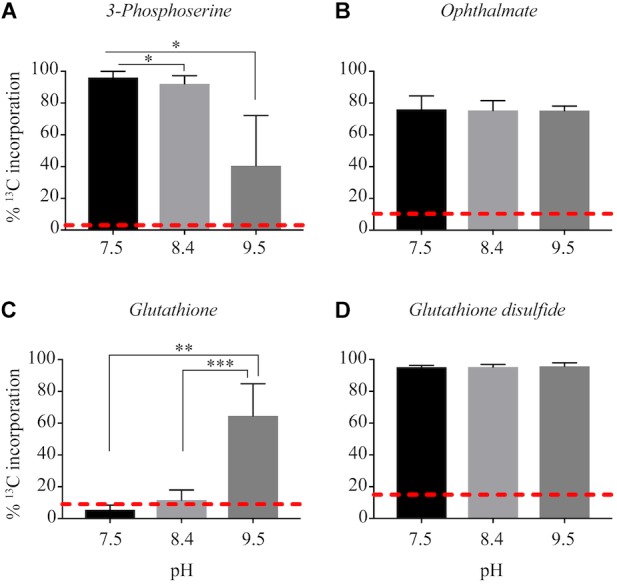
Incorporation of ^13^C from urea into other metabolites: 3-phosphoserine **(A)**, ophthalmate **(B)**, glutathione **(C)**, glutathione disulfide **(D).** Red dashed lines represent predicted natural ^13^C abundance, and error bars represent one standard error. Stars represent statistically significant differences in ^13^C incorporation percentages across pH treatments: ^∗^*p* < 0.01; ^∗∗^*p* < 0.05; ^∗∗∗^*p* < 0.001. All *p*-values generated from One-Way ANOVA can be found in Supplementary Table S3.

^13^C from urea was detected in multiple metabolites including amino acids, their precursors and metabolites involved in central C metabolism (Figure 4–6). Specifically, several metabolites with ^13^C signatures are key intermediates in the Calvin cycle, glycolysis and the pentose phosphate pathway (Figure 4A,C) as well as the arginine biosynthesis pathway (Figure 4B,C). In addition to arginine, other amino acids were observed to have ^13^C signatures, including glutamate, serine, aspartate, alanine, leucine, valine, lysine, threonine (Figure 4C, 5), and amino acid biosynthetic intermediates (Figure 6A). Notably, ^13^C was observed in all of the precursor amino acids for the production of microcystins. Some ^13^C enriched metabolites involved in oxidative stress had high percentages of incorporation such as glutathione (GSH), glutathione disulfide (GSSG) and ophthalmate (Figure 6B–D). ^13^C incorporation into GSSG was not different across varying pH, but GSH had little or undetectable incorporation at pH of 7.5 and 8.4, and an average incorporation of approximately 65% at pH of 9.5. ^13^C was incorporated into metabolites at all pH values, but metabolites from both central C metabolism and amino acid biosynthesis tended to be more enriched at high pH (Figure 4–6 and Supplementary Table S3). The addition of Na^+^ and NaHCO_3_ did not affect growth suggesting these cultures were not C starved in these conditions.

## Discussion

The factors that drive proliferation and success of cyanobacteria are numerous and poorly understood. While macronutrient enrichment (N and P) is a primary cause of blooms (Steffen et al., 2014a; Paerl et al., 2016), there are likely multiple factors contributing to how one or more species end up dominating an ecosystem. For example, the notorious *Microcystis* spp. persists under nutrient limitation by use of high-affinity transporters, production storage molecules for N and P, and use of organic sources of N and P (Harke et al., 2016). Their gas vacuoles allow for buoyancy in the water column to influence light exposure or scavenge nutrients, and they form colonies which may help to evade stress. They also prefer warmer water temperatures that are now being expanded and exacerbated by the changing climate (Guo, 2007; Paerl and Huisman, 2008; Harke et al., 2016; Xiao et al., 2018). All of these advantages help to overcome limitations or contribute to the use of nutrient elements, but it is ultimately C fixation or sequestration that dictates the success of phototrophs. Here, we used data generated from culture work using the model toxic cyanobacterium *M. aeruginosa* NIES843, real-time pH data and publicly accessible sequence information to demonstrate that urea can act as both a N and C source for this cyanobacterium, an ability that may partially account for the persistence (and perhaps even dominance) of *Microcystis* within freshwater blooms.

During algal bloom formation, an increase in pH is commonly observed (Paerl, 2018), as was seen in the western basin of Lake Erie during the 2015 *Microcystis* bloom (Figure 1B–G). Results from *M. aeruginosa* culture studies suggest not only that cyanobacteria are responsible for the rise in pH, but they indeed favor the more alkaline conditions often seen later in a bloom (Figure 1B,D,F). Cultures of *M. aeruginosa*, grown in buffered medium, did not always have higher growth rates, but the highest FSU values were seen at the higher pH values. This could indicate that higher pH supported a higher carrying capacity, or that these cells were more fluorescent and were in better photosynthetic health. A preference for slightly alkaline conditions is not a newly described phenomenon as cyanobacteria are typically cultured in these conditions, but the field data and culture work presented here suggested that the increase in the pH during a bloom serves as a positive feedback loop: algal blooms (like those dominated by *Microcystis* spp.) lead to increases in pH which lead to more prolific cyanobacterial growth. Changes in pH during the course of a bloom event impact DIC availability and composition, and this may have implications for species succession in lakes and other fresh waters since different *Microcystis* strains possess different mechanisms for C concentration and prefer different concentrations of CO_2(aq)_ (Sandrini et al., 2014; Sandrini et al., 2015). In addition, the success of *Microcystis* in alkaline conditions must be in part due to a competitive advantage against other phytoplankton (Hutchinson, 1961). For example, diatoms that bloom in early winter and spring in Lake Erie (Twiss et al., 2012; Hampton et al., 2017) require biogenic silica for their frustules (cell walls), but basic pH conditions can be corrosive to these protective structures (Lewin, 1961; Martin–Jézéquel et al., 2000) and decrease growth rate (Lundholm et al., 2004).

Growth rates of *M. aeruginosa* on varying N chemistries were also influenced by pH (Figure 2), implying N availability during the daily or seasonal alkalinity fluctuations is important. In contrast to nitrate and ammonium, elevated pH conditions on urea yielded the highest growth rates. An advantage or convenient coincidence of growth on urea as a N source may be that the inorganic C byproduct of urea hydrolysis can be used during times of C limitation or during conditions in which the DIC composition is unfavorable. However, much of the previous work on urea-C incorporation by phytoplankton has concluded that urea-C is released as a waste product (Mulholland et al., 2004; Jauzein et al., 2011); indeed the release of CO_2_ has been historically used as a method for measuring urease activity (Lund and Blackburn, 1989; Guettes et al., 2002). Where incorporation was observed, urea-C accounted for very little of the C uptake compared to HCO_3_ (Antia et al., 1977; Fan and Glibert, 2005). It is important to note that the assimilation of urea as a N source by heterotrophic bacteria in eutrophic systems would release CO_2_ and could confound these results. The importance of urea-C to phototrophs may be more relevant seasonally or in varying conditions (Andersson et al., 2006), such as the aforementioned change in the composition and decreased availability of inorganic C with increasing pH. The expression of *ureC* during a *Microcystis* bloom in Lake Erie indicates that urea was primarily used by cyanobacteria rather than heterotrophic bacteria (Supplementary Figure S6), implying urea-derived C would be assimilated rather than released during this prolific bloom that shutdown the Toledo water supply (Steffen et al., 2017).

Here, it was confirmed that *M. aeruginosa* can directly use urea as a supplemental C source in addition to serving as a N source. Cultures in this experiment were grown across a range of environmentally relevant pHs, capturing values observed across a typical bloom event, while still falling within the buffer capacity of the media. Notably, there were several metabolites with ^13^C signatures that are key intermediates in the Calvin cycle, pentose phosphate pathway and glycolysis, as well as amino acid biosynthesis regardless of pH. This implies that urea as a C source is not only valuable when dissolved inorganic C is primarily HCO_3_ and CO_3_; at a pH of 7.5 the inorganic C composition would be entirely HCO_3_ and CO_2_ and both chemistries can be utilized by *M. aeruginosa* (Supplementary Figure S1). This was possibly a result of the relatively low DIC concentrations in this media compared to other common fresh water media (i.e., BG-11) and CO_2(aq)_ saturated or hard water lakes (Casey et al., 1998; Visser et al., 2016). However, eutrophic lakes experience large fluctuations in DIC and range from CO_2(aq)_ super-saturated to CO_2(aq)_ deplete (Visser et al., 2016; Morales-Williams et al., 2017). Previously published transcriptomes (Steffen et al., 2014b) from cultures grown in similar conditions at a pH of 8.2 with the same low DIC concentrations showed that relevant CCMs were indeed active as expected: HCO_3_ was the dominant form of DIC at pH of 8.2 (Supplementary Figures S1, S7). Additions of Na^+^, to increase highly expressed SbtA pump, or HCO_3_ did not improve growth rates (Supplementary Figures S2, S8), suggesting the cells were not starved for C, but were only likely CO_2(aq)_-limited (*vis a vis* Eldridge et al., 2004). This indicated the benefit of urea may be greater under conditions in which CO_2(aq)_ specifically is low during dense blooms that change system pH and therefore, alkalinity and DIC composition. Interestingly, cells grown on urea had reduced expression of *sbtA* compared to cells grown on nitrate (Supplementary Figure S7), further supporting that urea is helping to satisfy C requirements. Although expression of *sbtA* was lower on ammonium than both urea and nitrate, this was likely reflective of the health of cells which were noted to have stunted growth (Steffen et al., 2014b), but still poses interesting questions about the interactions between N and C assimilation. Future studies will be needed to address the tradeoff between these two mechanisms of C acquisition with urea as the N source. However, the greater ^13^C incorporation percentages into several metabolites at pH 9.5 compared to 7.5 and 8.4 suggests that the pH driven composition change in DIC in more alkaline conditions influenced the utilization of urea-C. C was likely incorporated at higher rates with metabolites being more rapidly turned over, which is supported by higher growth rates at high pH and the general trend that metabolites were in lower abundances at the high pH (Figure 2, 3).

Interestingly, ^13^C from urea was also incorporated into the precursor amino acids necessary for microcystin production (Figure 5). Microcystin is a heptapeptide and a N- and C-rich molecule, and the influence of urea and other N forms on microcystin production have been, and continue to be, studied extensively (Davis et al., 2010; Gobler et al., 2016; Harke et al., 2016; Peng et al., 2018). Several studies provide evidence that urea is linked to microcystin production (Finlay et al., 2010; Donald et al., 2011; Harke et al., 2016). The incorporation of ^13^C from urea into the necessary precursors for microcystin suggests urea may serve as not only a N source for the production of this cyanotoxin, but also a C source. Indeed, ^13^C incorporation into some of these precursor amino acids was higher at high pH; however, the microcystin concentration was below our ability for detection in this study. Again, this may be a more significant observation at different times of the day or season and is a valuable consideration to make when sampling or performing experiments to address questions about N chemistry and microcystin production.

Another metabolite with higher ^13^C incorporation at pH 9.5 was GSH. At a pH of 9.5, approximately 60% of the total pool of GSH contained labeled C, whereas at the lower pH values ^13^C incorporation from urea was very little or completely absent. Total GSH abundance did not differ between treatments, which highlights the importance of this metabolite in *Microcystis* physiology regardless of pH. Although GSH is involved in several metabolic processes within the cell, in phototrophs it is an important antioxidant (Smirnova and Oktyabrsky, 2005; Cameron and Pakrasi, 2010; Cassier-Chauvat and Chauvat, 2014). In cyanobacteria, the ratio and concentrations of GSH (and its oxidized form, GSSG) help to balance the redox state and buffering capacity of the cell (Smirnova and Oktyabrsky, 2005). One of the benefits of CCMs and subsequent increased CO_2_ into the carboxysome of cyanobacteria is the contribution to reducing photoinhibitory effects under low CO_2(aq)_ conditions by inhibiting photorespiration (Tabita, 1994). A higher percentage of incorporation of ^13^C into GSH may be indicative that the C from urea is valuable to cells at a higher pH by reducing the cost to maintain a cellular redox balance, thereby promoting the growth and proliferation of cells.

The model toxic cyanobacterium *M. aeruginosa* possesses the genes necessary for the two mechanisms of urea assimilation (Supplementary Figure S9). The first reaction involves the catalyzed hydrolysis of urea to NH_4_^+^ and carbamate by the activity of the enzyme urease. Carbamate is unstable and thought to quickly dissociate into NH_4_^+^ and CO_2_ (Zimmer, 2000; Mikkelsen et al., 2010). Another mechanism of urea degradation, referred to as the elimination method, involves the uncatalyzed degradation of urea by urease and yields NH_4_^+^ and cyanate due to the activity of the enzyme cyanase (Krajewska, 2009). While this remains controversial (the elimination method has not been confirmed experimentally and only by *in silico* models) the end products would still be the same as hydrolytic decomposition of urea, 2NH_4_^+^ + CO_2_ (Krajewska, 2009). Although the details of this mechanism for assimilation are not clear, incorporation of ^13^C from urea into intermediates within the Calvin cycle would suggest the point of entry of C from urea is through C-fixation (Figure 4). Alternatively, another possible entry point of urea-C exists through activity of carbamoyl phosphate synthetase (CPS, Kim and Raushel, 2004). CPS can phosphorylate carbamate, the intermediate in urea hydrolysis, to yield carbamoyl phosphate (carbamoyl-P) and this directly feeds into the arginine biosynthesis pathway, which was enriched in ^13^C in this study (Figure 4 and Supplementary Figure S9). *M. aeruginosa* NIES843 carries genes for the large and small subunits of CPS (MAE_RS21880, CarB; MAE_RS12410, CarA). Carbamate, albeit short-lived, is already a reduced form of C, and it would be an energetically favorable mechanism of assimilation of the C from urea compared to undergoing C fixation (which requires both ATP and reducing power). This mechanism could also serve as an entry point for N into cellular biochemistry and suggests greater production of arginine, which is an important precursor for cyanophycin, a N storage molecule, and microcystin. While this hypothesis needs to be experimentally confirmed, this may have implications regarding the persistence and toxicity of *Microcystis* when urea is present in the environment. Indeed, *carB,* the large subunit of CPS, was expressed by *Microcystis* during the 2014 bloom, while also expressing genes for urea uptake and utilization (Steffen et al., 2017). Utilization of *carA/carB* mechanisms for C assimilation may not be restricted to phototrophs in these systems as these genes are widely spread among heterotrophic bacteria as well, allowing microbes to capitalize on the urea as a readily available C source via this path. However, re-examination of Lake Erie metatranscriptomes from 2014 indicates *carB* expression was primarily from cyanobacteria (Supplementary Figure S6). In conjunction with the observation that almost all of the *ureC* expression was from cyanobacteria, this suggests that the mechanism of entry for C would be environmentally relevant.

Regardless of the mechanism for urea-C assimilation, growing on urea may be cost effective to a cell under C-limitation, as inorganic C is “freely” given. Assuming a “Redfield ratio” of C:N of 7:1 as a conserved quota for *Microcystis* cells (Redfield, 1958), growth on urea could support 7.6% of a cell’s C requirements. Thus, while not supplying the total C needs of a cell, urea may be particularly important during periods of the day where conditions are prime for growth (sufficient light, higher temperatures) yet dissolved CO_2_ has become vanishingly low due to physiochemical conditions (i.e., elevated pH) and competition. Indeed, the CCMs are active in low DIC conditions when growing on urea (Supplementary Figure S7) suggesting that urea-C would be supplementing the C requirement of the cells.

The use of urea as a N fertilizer in agriculture has increased rapidly over the past several decades, and its role as a potential driver of eutrophication is important as it is commonly found in freshwater systems (Glibert et al., 2006). In this study, we have confirmed urea can serve as a N source to *Microcystis* spp. and is a preferred source of N for growth at high pH values commonly observed during blooms. Moreover, we show urea can be used as a source of C for *M. aeruginosa,* regardless of the availability of other usable forms of inorganic C. It is likely there is a greater need for C from urea at higher pH values, when dissolved CO_2_ in the water is absent, and this supplemental C may aid in managing the redox state and contribute to the success of *Microcystis* for prolonged periods of time. We propose that urea can be an energetically favorable C source that helps support metabolic homeostasis and photosynthetic efficiency of *Microcystis* during dense blooms. While this physiological mechanism may not fully explain how *Microcystis* manages to dominate planktonic communities, it does add an intriguing and relevant piece to the puzzle.

## Author Contributions

LK and BZ performed the cultivation experiments. LK performed the ^13^C experiment and analyzed the environmental metadata, environmental metatranscriptomes, and culture transcriptomes. LK and AF extracted the metabolites. AF, HC, and SC performed the metabolomics. LK, SW, HC, BZ, and SC drafted the manuscript. All authors contributed to the metabolomic data analysis and study design.

## Conflict of Interest Statement

The authors declare that the research was conducted in the absence of any commercial or financial relationships that could be construed as a potential conflict of interest.

## Footnotes

1 https://www.mg-rast.org/index.html
