# Supplementary material for: Urea Is Both a Carbon and Nitrogen Source for Microcystis aeruginosa: Tracking 13C Incorporation at Bloom pH Conditions
Source: Front Microbiol. 2019 May 17;10:1064. doi: 10.3389/fmicb.2019.01064 (PMC6536089; doi:10.3389/fmicb.2019.01064)
Supplement: Supplementary file 2 [file Data_Sheet_1.pdf]

FRONTERS IN MICROBIOLOGY

TITLE: Urea is both a carbon and nitrogen source for *Microcystis aeruginosa*: tracking ^{13}C incorporation at bloom pH conditions

SUPPLEMENTAL MATERIAL

AUTHORS:
Lauren E. Krausfeldt[1], Abigail T. Farmer[2], Hector F. Castro Gonzalez[2], Brittany N. Zepernick[1], Shawn R. Campagna[2], and Steven W. Wilhelm[1] *

[1.] Department of Microbiology, University of Tennessee Knoxville, TN
[2.] Department of Chemistry, University of Tennessee, Knoxville, TN

*author for correspondence: wilhelm@utk.edu

Table S1. Cell concentration and chlorophyll *a* autofluorescence (FSU) at time of sample collection for the labeling experiments.

Sample	Cells/mL	FSU
**7.5 R1**	146,696	190.3
**7.5 R2**	144,999	179.7
**7.5 R3**	139,071	167.9
**8.4 R1**	321,374	225.4
**8.4 R2**	86,493	36.02
**8.4 R3**	413,806	291.4
**9.5 R1**	927,852	362.7
**9.5 R2**	986,132	379.1
**9.5 R3**	1,293,915	419.7

Table S2. Adjusted p values derived from Tukey's multiple comparison test after pairwise comparisons of growth rates between each N and pH treatment. In bold are statistically significant ($p < 0.05$) values.

Comparisons	Adjusted P Value	Comparisons	Adjusted P Value
7.7:NO3 vs. 7.7:NH4	0.4394	8.2:NO3 vs. 8.7:NH4	>0.9999
7.7:NO3 vs. 7.7:Urea	0.0939	**8.2:NO3 vs. 8.7:Urea**	**<0.0001**
7.7:NO3 vs. 8.2:NO3	>0.9999	8.2:NO3 vs. 9.2:NO3	0.9992
**7.7:NO3 vs. 8.2:NH4**	**<0.0001**	8.2:NO3 vs. 9.2:NH4	>0.9999
**7.7:NO3 vs. 8.2:Urea**	**<0.0001**	**8.2:NO3 vs. 9.2:Urea**	**<0.0001**
7.7:NO3 vs. 8.7:NO3	>0.9999	**8.2:NH4 vs. 8.2:Urea**	**0.0007**
7.7:NO3 vs. 8.7:NH4	>0.9999	**8.2:NH4 vs. 8.7:NO3**	**<0.0001**
**7.7:NO3 vs. 8.7:Urea**	**<0.0001**	**8.2:NH4 vs. 8.7:NH4**	**<0.0001**
7.7:NO3 vs. 9.2:NO3	>0.9999	**8.2:NH4 vs. 8.7:Urea**	**0.0099**
7.7:NO3 vs. 9.2:NH4	>0.9999	**8.2:NH4 vs. 9.2:NO3**	**<0.0001**
**7.7:NO3 vs. 9.2:Urea**	**<0.0001**	**8.2:NH4 vs. 9.2:NH4**	**<0.0001**
**7.7:NH4 vs. 7.7:Urea**	**0.0004**	**8.2:NH4 vs. 9.2:Urea**	**0.0035**
7.7:NH4 vs. 8.2:NO3	0.7655	**8.2:Urea vs. 8.7:NO3**	**<0.0001**
**7.7:NH4 vs. 8.2:NH4**	**<0.0001**	**8.2:Urea vs. 8.7:NH4**	**<0.0001**
**7.7:NH4 vs. 8.2:Urea**	**0.0001**	8.2:Urea vs. 8.7:Urea	>0.9999
7.7:NH4 vs. 8.7:NO3	0.1617	**8.2:Urea vs. 9.2:NO3**	**<0.0001**
7.7:NH4 vs. 8.7:NH4	0.886	**8.2:Urea vs. 9.2:NH4**	**<0.0001**
**7.7:NH4 vs. 8.7:Urea**	**0.0002**	8.2:Urea vs. 9.2:Urea	>0.9999
7.7:NH4 vs. 9.2:NO3	0.3391	8.7:NO3 vs. 8.7:NH4	0.9881
7.7:NH4 vs. 9.2:NH4	0.796	**8.7:NO3 vs. 8.7:Urea**	**<0.0001**
**7.7:NH4 vs. 9.2:Urea**	**<0.0001**	8.7:NO3 vs. 9.2:NO3	>0.9999
**7.7:Urea vs. 8.2:NO3**	**0.032**	8.7:NO3 vs. 9.2:NH4	0.9973
**7.7:Urea vs. 8.2:NH4**	**<0.0001**	**8.7:NO3 vs. 9.2:Urea**	**<0.0001**
**7.7:Urea vs. 8.2:Urea**	**<0.0001**	**8.7:NH4 vs. 8.7:Urea**	**<0.0001**
7.7:Urea vs. 8.7:NO3	0.2714	8.7:NH4 vs. 9.2:NO3	0.9986
**7.7:Urea vs. 8.7:NH4**	**0.044**	8.7:NH4 vs. 9.2:NH4	>0.9999
**7.7:Urea vs. 8.7:Urea**	**<0.0001**	**8.7:NH4 vs. 9.2:Urea**	**<0.0001**
7.7:Urea vs. 9.2:NO3	0.2644	**8.7:Urea vs. 9.2:NO3**	**<0.0001**
**7.7:Urea vs. 9.2:NH4**	**0.0658**	**8.7:Urea vs. 9.2:NH4**	**<0.0001**
**7.7:Urea vs. 9.2:Urea**	**<0.0001**	8.7:Urea vs. 9.2:Urea	>0.9999
**8.2:NO3 vs. 8.2:NH4**	**<0.0001**	9.2:NO3 vs. 9.2:NH4	0.9998
**8.2:NO3 vs. 8.2:Urea**	**<0.0001**	**9.2:NO3 vs. 9.2:Urea**	**<0.0001**
8.2:NO3 vs. 8.7:NO3	0.9901	**9.2:NH4 vs. 9.2:Urea**	**<0.0001**

Table S3. Adjusted p values derived from Tukey's multiple comparison tests after pairwise comparisons between percentages of ^{13}C incorporation into metabolites at different pH. In bold are statistically significant ($p < 0.10$) values.

	7.5 vs 8.4	7.5 vs 9.5	8.4 vs 9.5
Glyceraldehyde -3P	*0.9752*	*0.1899*	*0.3129*
Glucose 6P	*0.4604*	***0.0260***	*0.1658*
Glycerone-P	*0.9468*	*0.1776*	*0.3315*
Sedoheptulose-1,7BP	*0.2915*	***0.0004***	***0.0019***
Glutamate	*0.1189*	***0.0333***	*0.7298*
Glutamine	*0.6267*	***0.0294***	*0.1295*
N-acetylglutamate	***0.0557***	***0.0095***	*0.4498*
Aspartate	***0.0342***	***0.0010***	***0.0287***
Arginine	***0.0515***	*0.5862*	*0.1467*
Alanine	*0.9884*	*0.6885*	*0.8165*
Serine	*0.885*	*0.1108*	*0.2625*
Leucine	*0.3823*	*0.2576*	*0.9861*
Threonine	*0.7530*	*0.1061*	*0.3406*
3-phosphoserine	*0.9762*	***0.0501***	***0.0923***
Glutathione	*0.8902*	***0.0093***	***0.0228***
Glutathione Disulfide	*0.9995*	*0.9407*	*0.9610*
Valine	*0.2678*	***0.0108***	***0.1012***

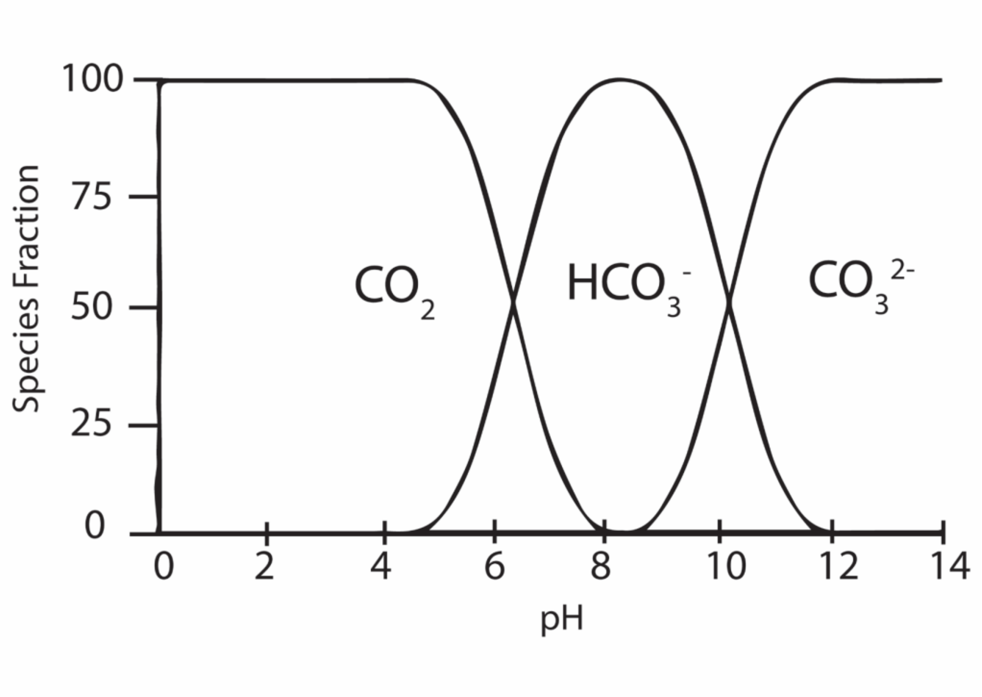

Figure S1. Inorganic carbon speciation in freshwater systems in relation to pH (figure redrawn and adapted from Wetzel, 2001).

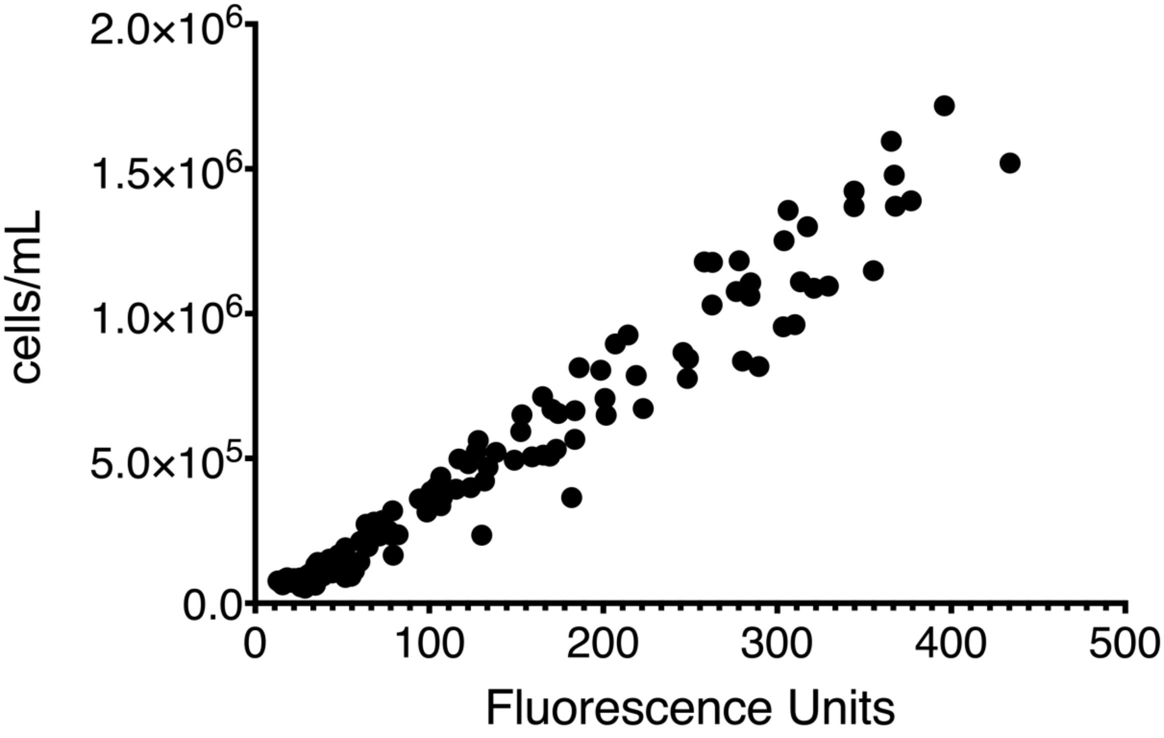

Figure S2. The relationship between cell concentration and chlorophyll *a* autofluorescence for *Microcystis aeruginosa* NIES843. Cells were grown for 7-10 days in CT medium with either nitrate, urea or ammonia at pH of 7.7, 8.2, 8.7 or 9.2 in a replicative manner in conditions previously described in the methods section for the pH growth curves. Pearson $R^2 = 0.96$, $p < 0.0001$.

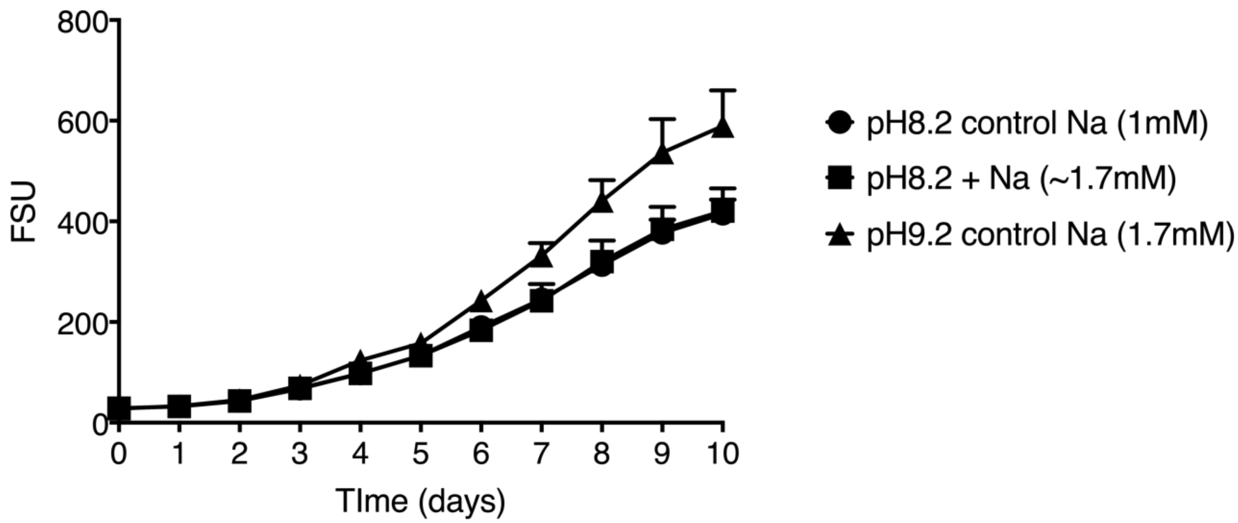

Figure S3. Growth of *Microcystis aeruginosa* NIES843 on CT medium with 0.595 mM N as nitrate. NaCl was added to media at a pH of 8.2 so that total Na concentration matched media at pH of 9.2 that was adjusted with a larger amount of NaOH. Cultures were inoculated at an FSU (chlorophyll *a* autofluorescence) of ~25 and incubation conditions were as previously described. FSU was measured daily at approximately the same time every day.

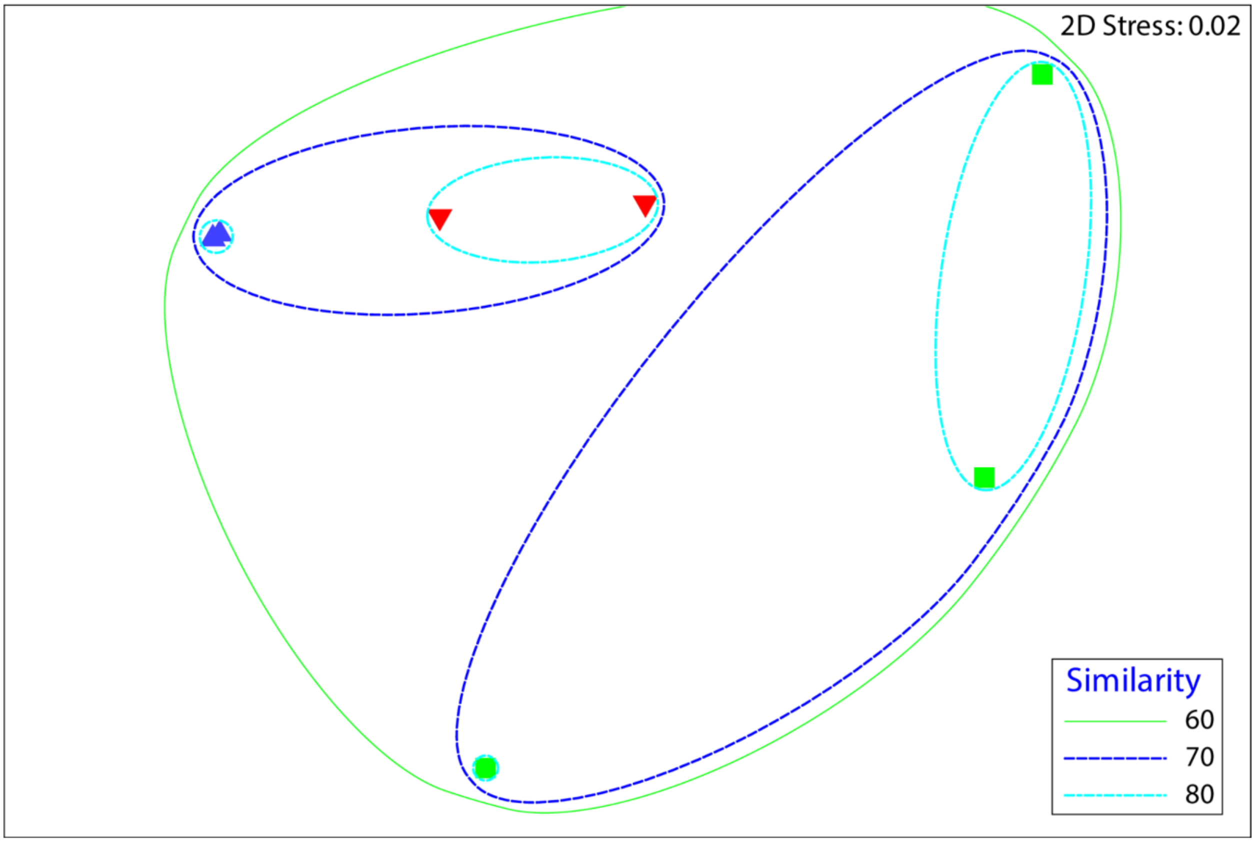

Figure S4. nMDS describing relationships between metabolites from *M. aeruginosa* NIES843 when growing at different pH. Total abundances for metabolites were normalized by cell number, log(x+1) transformed and clustered using Bray-Curtis similarity. 2D stress = 0.02; blue = pH 7.5; red = pH 8.4; green = pH 9.5

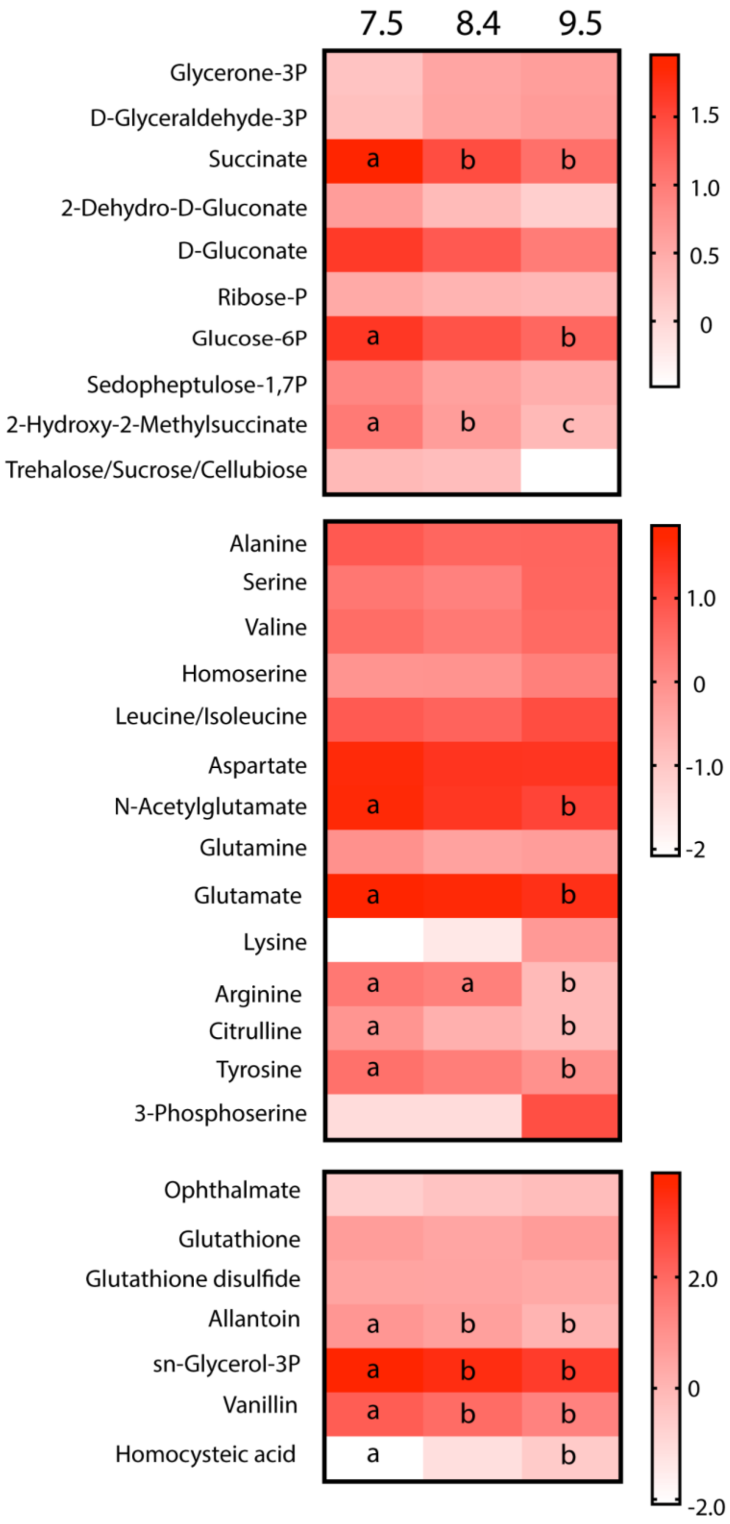

Figure S5. Heatmap comparing relative abundances of metabolites between the different pH treatments. Abundances were square root transformed for visualization. Different letters represent statistically different abundances (<0.05) in metabolites.

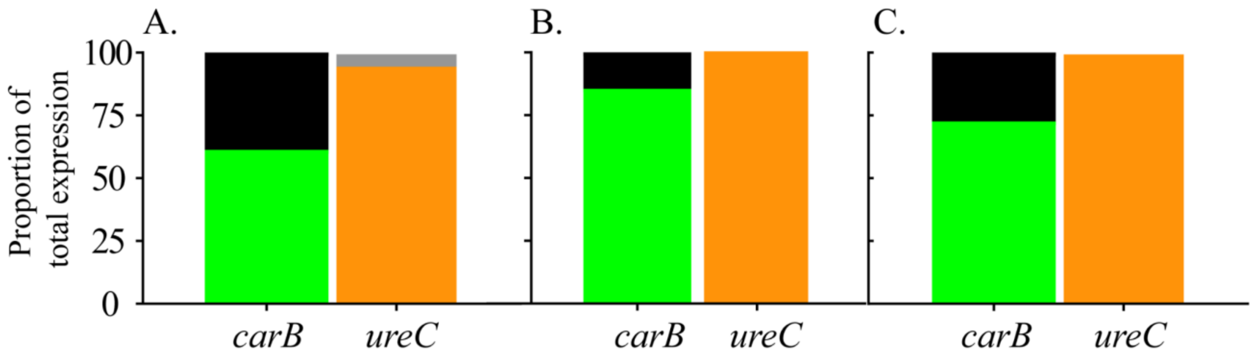

Figure S6. Proportional expression of *carB* and *ureC* by cyanobacteria (green and orange, respectively) and "other" members of the microbial community (black and grey, respectively) at stations WE2 (A), WE4 (B) and WE8 (C) in the Western basin of Lake Erie 2014.

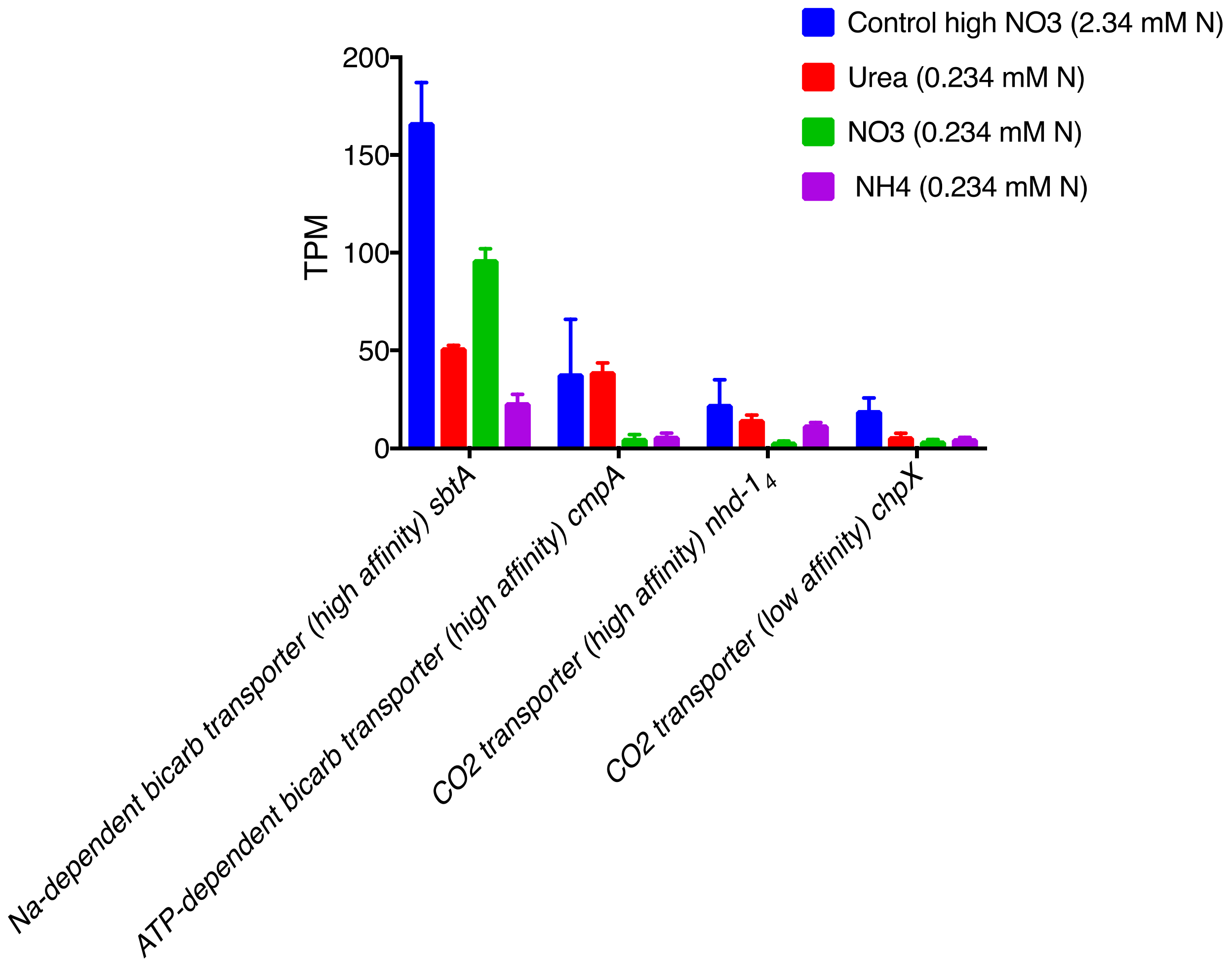

Figure S7. Expression of genes (transcripts per million, TPM) involved in carbon concentrating mechanisms in *M. aeruginosa* NIES843 in CT medium grown with different N sources from previously a published study (Steffen et al., 2014b). Genes for this analysis were identified from Sandrini et al., 2014.

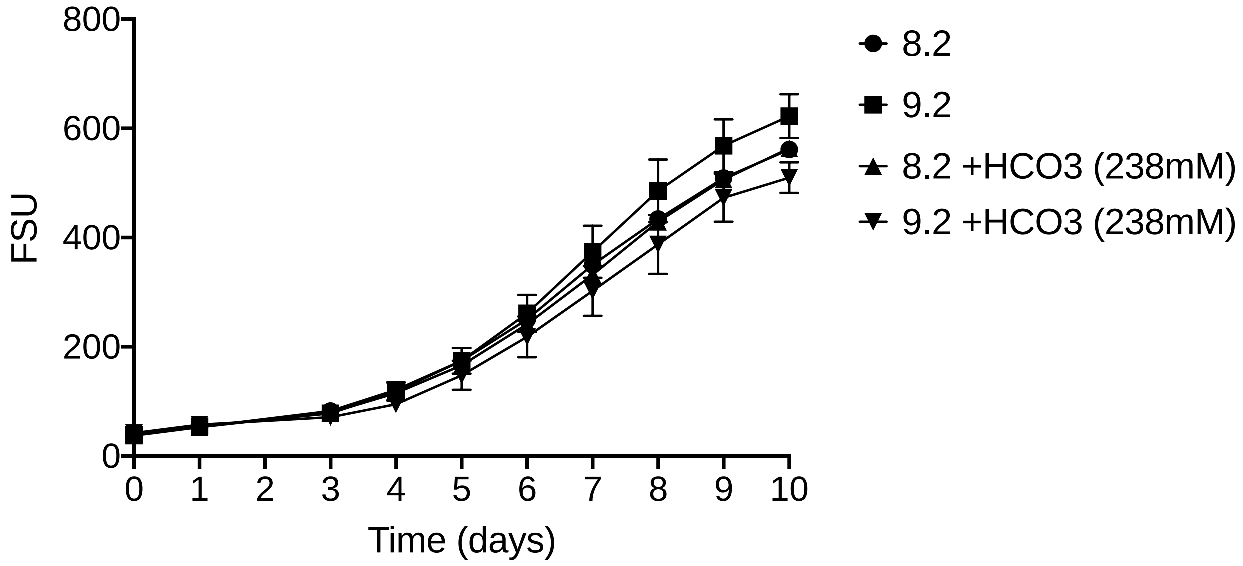

Figure S8. Growth of *M. aeruginosa* NIES843 with additions of sodium bicarbonate. Cells harvested in mid-log phase were inoculated into CT media at a pH of 8.2 or 9.2 with and without the addition of bicarbonate at a concentration comparable to other fresh water media (BG11). Chlorophyll a autofluorescence was measured daily at approximately the same time every day, and other incubation conditions were as previously described.

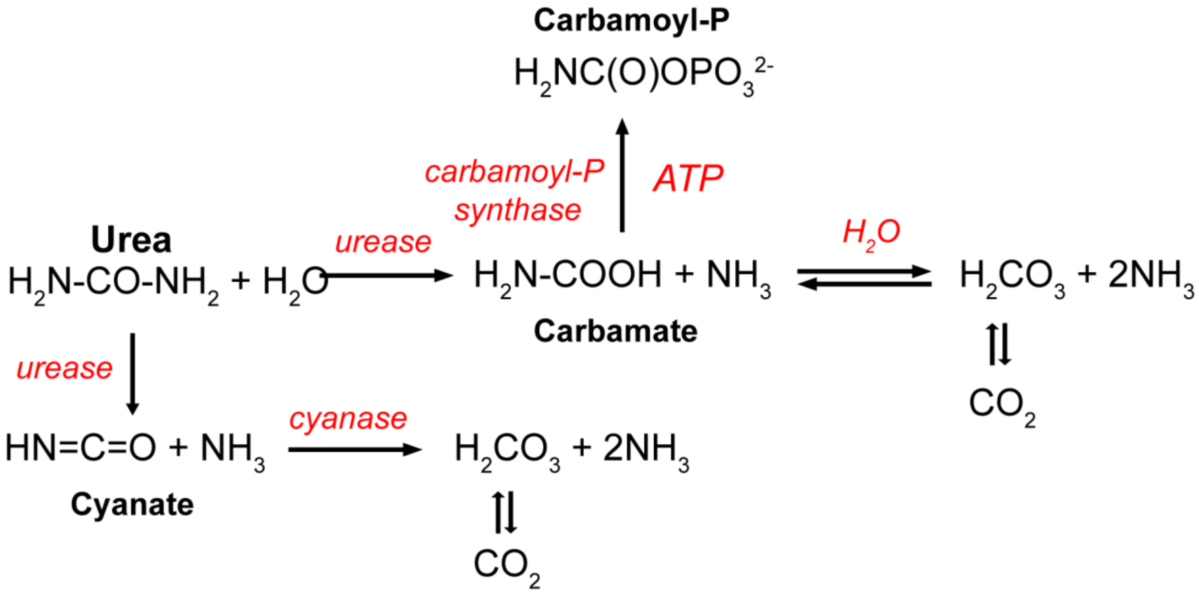

Figure S9. Proposed pathways of urea degradation and CO_2 assimilation by *M. aeruginosa* NIES843.
